# Supplementary material for: Evaluating the long-term operational performance of a large-scale inland terminal: A discrete event simulation-based modeling approach
Source: PLoS One. 2022 Dec 5;17(12):e0278649. doi: 10.1371/journal.pone.0278649 (PMC9721477; doi:10.1371/journal.pone.0278649)
Supplement: S1 Appendix — (DOCX) [file pone.0278649.s001.docx]

**Appendix A. A list of abbreviations**

CHE Container handling equipment

DES Discrete event simulation

ESCO Eastern Sea Laem Chabang Terminal Co., Ltd.

FCL Full container load

LCL Less than container load

LICD Ladkrabang Inland Container Depot

PAT Port Authority of Thailand

RS Reach stacker

RTG Rubber tyred gantry crane

SRT State Railway of Thailand

TEU Twenty-foot equivalent unit

TL Top loader

YT Yard truck
