# Supplementary material for: Evaluating the long-term operational performance of a large-scale inland terminal: A discrete event simulation-based modeling approach
Source: PLoS One. 2022 Dec 5;17(12):e0278649. doi: 10.1371/journal.pone.0278649 (PMC9721477; doi:10.1371/journal.pone.0278649)
Supplement: Attachment — Submitted filename: Response to Reviewers Comments - R2.pdf [file pone.0278649.s003.pdf]

Response to Reviewer 1

We would like to thank the reviewer for valuable comments, which help improve the quality of our manuscript. In the following, we provide an overview of changes we have made according to the comments and suggestions. We will include our responses below each of the reviewer's comments (which are included in blue).

1. The contribution of the paper is merely local cases of LICD.

- We would like to reiterate that the investigation of Ladkrabang Inland Container Depot (LICD), in this present work, is only an example demonstrating the usefulness of Discrete Event Simulation (DES) framework, which could be extended or, even, applied to other complex systems — including manufacturing or logistics-related systems discussed in Section 1 (Lines 75 – 78) and Section 5 (Lines 636 – 640).

    - Section1: Introduction (Lines 75 – 78)

      "It could be seen from the previous research that simulation has played an important role in the evaluation of inland/coastal terminal performance [25-28], as well as that of other logistics-related facilities [29,30], whose detailed settings may vary depending on the investigated operations and platforms."

    - Section 5: Discussion and conclusions (Lines 636 – 640)

      "Despite assumptions made to simplify the whole system, we expect that our proposed DES models would be found useful to relevant players in the logistical industry, as the models could be applied in the performance evaluation of similar facilities. They could also be extended for use in the investigation of multimodal transportation networks with multiple types of facilities and modes of transportation — which is worth exploring in subsequent papers."

- Although the devised DES model has been specifically constructed for the analyses of LICD, such a modeling framework could be extended to other dry ports or similar logistics-related facilities with the same or different CHE types; but this may require modifications on the model's (sub)modules, as well as those on the operational rules of investigated facilities (it is less likely that one simulation model would fit all of the existing dry ports across the globe).

  In order to avoid any confusion that might arise, the objectives – and so the practical contributions of this paper – are clearly emphasized in Lines 88 – 103.

    - Section1: Introduction (Lines 88 – 103)
      "In light of this gap, this paper aims to build a more realistic DES model that takes into account not only the operations of a dry port with shared CHE but also other managerial factors that could potentially affect a dry port's long-term performance, including infrastructure, multimodal transportation policies, and the future course of a dry port from the regulator's point of view. For ease of discussion, the proposed DES model will be developed based on the Ladkrabang Inland Container Depot

> (LICD) — the first and only containerized dry port in Thailand — and its development plan through the help of SIMIO simulation program. With this proposed model, related players should be able to accurately evaluate the current dry port's performance, as well as that of a new layout at various development stages prior to the implementation. The proposed model will also allow the players to conduct detailed analyses under different scenarios, which will, in turn, help enhance the concurrent utilization of expensive CHE, the dry port, and the respective multimodal transportation network. It is worth remarking that, while the devised DES model has been specifically constructed for the analyses of LICD, such a modeling framework could be extended to other dry ports or similar logistics-related facilities with the same or different CHE types; but this may require modifications on the model's (sub)modules, as well as those on the operational rules of investigated facilities."

2. The description of the methodology is still unclear.

- As mentioned in the previous rebuttal letter that the simulation models representing LICD operations are rather complex, for ease of discussion/presentation, we therefore need to divide such models into several subsystems and discuss each of them in great detail in Section 3. In case that the readers would like to see the detailed description of such models, we invite them to visit the public repository that the complete simulation models have been placed (https://github.com/ORCChula/LICD).

- Please note that all models in the repository are ready for execution. Interested readers may then download and further modify them for their specific purposes.

3. All figures are of low quality, and they lack an important point of the figure.

- We would like to reiterate that all figures are generated according to the Journal's standard (we also double checked the quality of all figures by PACE, as suggested by PLOS ONE). Furthermore, all figures are provided to help readers comprehend the operational flow of all subsystems.

In addition to the aforementioned responses, there are also many small changes that we have made to better improve the quality of this manuscript. We do hope that our explanation is clear; and, if further discussion is needed, we would be more than pleased to give clarification.

Response to Reviewer 2

We would like to thank the reviewer for valuable comments, which help improve the quality of our manuscript.

Response to Reviewer 3

We would like to thank the reviewer for valuable comments, which help improve the quality of our manuscript. In the following, we provide an overview of changes we have made according to the comments and suggestions. We will include our responses below each of the reviewer's comments (which are included in blue).

1. The abstract should be improved and must be cleared, especially the result.

- We do appreciate the reviewer's comment in this regard. The abstract has been accordingly revised to emphasize the results of this study.

2. I would also suggest the authors reduce the number of abbreviations along the manuscript.

- We do agree with the reviewer that there are too many abbreviations, which are difficult to follow. We have accordingly reduced (or even removed) the use of such abbreviations (when applicable); and, the following is an updated list of abbreviations.
    - Appendix A. A list of abbreviations (Lines 641 – 654)

"CHE	Container handling equipment
DES	Discrete event simulation
ESCO	Eastern Sea Laem Chabang Terminal Co., Ltd.
FCL	Full container load
LCL	Less than container load
LICD	Ladkrabang Inland Container Depot
PAT	Port Authority of Thailand
RS	Reach stacker
RTG	Rubber tyred gantry crane
SRT	State Railway of Thailand
TEU	Twenty-foot equivalent unit
TL	Top loader
YT	Yard truck"

In addition to the aforementioned responses, there are also many small changes that we have made to better improve the quality of this manuscript. We do hope that our explanation is clear; and, if further discussion is needed, we would be more than pleased to give clarification.
