# Supplementary material for: Evaluating the long-term operational performance of a large-scale inland terminal: A discrete event simulation-based modeling approach
Source: PLoS One. 2022 Dec 5;17(12):e0278649. doi: 10.1371/journal.pone.0278649 (PMC9721477; doi:10.1371/journal.pone.0278649)
Supplement: Attachment — Submitted filename: Response to Reviewers Comments.docx [file pone.0278649.s002.docx]

**Response to Reviewer 1**

We would like to thank the reviewer for valuable comments, which help improve the quality of our manuscript. In the following, we provide an overview of changes we have made according to the comments and suggestions. We will include our responses below each of the reviewer’s comments (which are included in blue).

1. Lack of novelty and contribution, the author only discusses the results from the Ladkrabang Inland Container Depot (LICD) case with no significant impact on the current development of container terminal efficiency for example automatization, double stack crane, etc.

- We do understand the concern raised by the reviewer in this regard; and, we would like to use this opportunity to address the reviewer’s concern. To begin with, this paper’s main objective is not to devise a new solution methodology but rather to develop a proper framework for the performance evaluation of large-scale dry ports based on the existing tools. The main contributions of this paper are, therefore, practical, which are evident from our findings that potentially support the development of dry ports, as well as that of similar logistics-related facilities, in a more sustainable fashion.
- It should be remarked that the investigation of Ladkrabang Inland Container Depot (LICD), in this present work, could be regarded as a success example demonstrating the applicability of our proposed modeling framework. And, surely, we can extend such a modeling framework to include the use of other systems (*e.g.,* automatization or double stack crane) — although some modifications are in need so that the constructed models well represent the investigated systems.
- We have to apologize the reviewer that the aforementioned facts may not well emphasized in the previous manuscript, which eventually leads to this confusion. To address the review’s concern, parts of the manuscript have been accordingly revised to emphasize the research objective, together with the applicability of the proposed modeling framework, as follows.
  - Introduction (Lines 85 – 100)

“In light of this gap, this paper aims to build a more realistic DES model that takes into account not only the operations of a dry port with shared CHE but also other managerial factors that could potentially affect a dry port’s long-term performance, including infrastructure, multimodal transportation policies, and the future course of a dry port from the regulator’s point of view. For ease of discussion, the proposed DES model will be developed based on the Ladkrabang Inland Container Depot (LICD) — the first and only containerized dry port in Thailand — and its development plan through the help of SIMIO simulation program. With this proposed model, related players should be able to accurately evaluate the current dry port’s performance, as well as that of a new layout at various development stages prior to the implementation. The proposed model will also allow the players to conduct detailed analyses under different scenarios, which will, in turn, help enhance the concurrent utilization of expensive CHE, the dry port, and the respective multimodal transportation network. It is worth remarking that, while the devised DES model has been specifically constructed for the analyses of LICD, such a modeling framework could be extended to other dry ports or similar logistics-related facilities with the same or different CHE types; but this may require modifications on the model’s (sub)modules, as well as those on the operational rules of investigated facilities.”

- Discussion and conclusions (Lines 590 – 593)

“In order to properly assess the operational performance of a dry port while taking into consideration both internal and external factors, a discrete event simulation (DES) framework has been herein developed using SIMIO simulation platform and applied to the Ladkrabang Inland Container Depot (LICD) — the first and only inland terminal in Thailand.”

- Discussion and conclusions (Lines 631 – 635)

“Despite assumptions made to simplify the whole system, we expect that our proposed DES models would be found useful to relevant players in the logistical industry, as the models could be applied in the performance evaluation of similar facilities. They could also be extended for use in the investigation of multimodal transportation networks with multiple types of facilities and modes of transportation — which is worth exploring in subsequent papers.”

2. The description of the methodology is unclear, no detailed algorithm has been explained, and the author only displays the DES model taken from the software used instead of writing the pseudocode.

- We have to apologize the reviewer that the description of the methodology — especially that related to SIMIO simulation program — is not well stated in the methodology section, which eventually leads to this misunderstanding. Actually, SIMIO is a program that helps simulate the system; but, the users/developers still need to design and transform the underlying systems into proper simulation models that well represent the systems on their own (*i.e.,* SIMIO does not provide ready-to-use packages for all systems).
- Since the simulation models representing LICD operations are rather complex, we therefore divide such models into several subsystems and discuss each of them in great detail in Section 3.2. And, for ease of understanding, the flowchart for each subsystem is thence provided (instead of a less practical detailed algorithm) to help readers comprehend the operational flow of such a subsystem (please note that the flowcharts are not the products of SIMIO).
- In order to address the reviewer’s concern, we have decided to place the simulation models that we construct in the public repository, alongside the datasets, so that interested readers could download, study, and further modify them for their specific purposes.

3. All figures are presented in low quality and not in a good manner of high-impact publication.

- Thank you for pointing this out. Regarding the quality of figures, we have ensured that all figures are generated according to the Journal’s standard (we also double checked the quality of all figures by PACE, as suggested by PLOS ONE).
- Please note that, although the quality of figures seems a bit off in the generated document, they are all in good shape if downloaded. We expect that the problem may lie in the process of document generation by Editorial Manager; and, we have already contacted the editorial office regarding this issue in our previous manuscript submission.

4. There are no field measurements to validate the simulation results.

- We do agree with the reviewer that the results and analysis section could be further improved if we have up-to-date data of the current LICD systems/operations. However, as the LICD is currently undergoing a transformation in terms of infrastructure — and so its operations — the data collected during this transient state would be not that useful, but time consuming.
- Yet, the constructed models have been verified and validated to ensure that they indeed represent the LICD based on previous datasets, as indicated in Lines 390 – 392.
  - Lines 390 – 392

“Prior to conducting a simulation, the proposed DES models have been initially assessed based on the results of simulation trials so that we could minimize the margin of error while ensuring that no desirable states, such as deadlocks, would be encountered during simulation runs.”

In addition to the aforementioned responses, there are also many small changes that we have made to better improve the quality of this manuscript. We do hope that our explanation is clear; and, if further discussion is needed, we would be more than pleased to give clarification.

**Response to Reviewer 2**

We would like to thank the reviewer for valuable comments, which help improve the quality of our manuscript. In the following, we provide an overview of changes we have made according to the comments and suggestions. We will include our responses below each of the reviewer’s comments (which are included in blue).

1. The authors used too many abbreviations, such as CHE, LICD, SRT, BKR, PAT, YT, TL, ESCO, etc., that make the text difficult to read and especially for people that are not so close with this matter. Please try to reduce them.

- We do agree with the reviewer that there are too many abbreviations, which are difficult to follow. We have accordingly reduced the use of such abbreviations and provided a list of abbreviations in Appendix A (Lines 636 – 653).
  - Appendix A. A list of abbreviations (Lines 636 – 653)

“BKP	Bangkok Port

CHE	Container handling equipment

DES	Discrete event simulation

ESCO	Eastern Sea Laem Chabang Terminal Co., Ltd.

FCL	Full container load

LCL	Less than container load

LCP	Laem Chabang Port

LICD	Ladkrabang Inland Container Depot

NT	North terminal

PAT	Port Authority of Thailand

RS	Reach stacker

RTG	Rubber tyred gantry crane

SRT	State Railway of Thailand

ST	South terminal

TEU	Twenty-foot equivalent unit

TL	Top loader

YT	Yard truck”

2. In my opinion, in the abstract should be added some text about DES (Discrete Event Simulation) and why the authors choose it in their research as the only one appropriate method.

- We do appreciate the reviewer’s comment in this regard. The abstract has been accordingly revised to emphasize the discrete event simulation and its advantages.

3. The list with references should be done in alphabetical order.

- We do understand the reviewer that many journals have adopted the alphabetical order method for citation. But, according to PLOS ONE’s requirements, the citation should follow the numbered citation (citation-sequence) method.

4. I recommend the authors to check once again the spelling and grammar in English before sending the revised manuscript. Some phrases (for clarity, unlike) and words (incremental) have to be replaced with appropriate synonyms.

- Thank you for pointing this out. The manuscript has been proofread by a proofreading service before submission, in order to avoid any plausible grammatical errors and typos.

In addition to the aforementioned responses, there are also many small changes that we have made to better improve the quality of this manuscript. We do hope that our explanation is clear; and, if further discussion is needed, we would be more than pleased to give clarification.
